# Report of the Committee of Selection

**Published:** 1920-07-17

**Authors:** 


### OUTDOOR MEDICAL STAFF OF THE MINISTRY OF HEALTH.

### Report of the Committee of Selection.

The Committee appointed on March 24, 1920, to assist the Minister of Health in the selection of suitable candidates for the posts of Medical Officer of the Ministry has now issued its Report, which is signed by all the members—viz.:—

* T. W. Shore, M.D., M.R.C.S., L.R.C.P. (Chairman), Dean of the Medical School, St. Bartholomew's Hospital; *E. Collingwood Andrews, M.D., B.Sc., Consulting Medical Officer to the Hampstead Maternity Home; *Sir Hamilton A. Ballance, K.B.E., C.B., M.S., F.R.C.S., Surgeon, Norfolk and Norwich Hospital; Professor R. A. Bolam, O.B.E., M.D., M.R.C.P., Hon. Physician, Skin Department, Royal Infirmary, Newcastle-on-Tyne, Professor of Medical Jurisprudence, University of Durham; H. G. Dain, M.B., M.R.C.S., L.R.C.P., Chairman of the Birmingham Insurance Committee; Professor Arthur J. Hall, M.D., F.R.C.P., Senior Physician, Sheffield Royal Hospital, Professor of Medicine, University of Sheffield; Sir Walter Kinnear, K.B.E., Controller of National Health Insurance, Ministry of Health; E. J. Maclean, M.D., M.R.C.P., Senior Gynecologist, Cardiff Infirmary, Lecturer on Midwifery, University College, S. Wales; J. Smith Whitaker, M.R.C.S., L.R.C.P., Senior Medical Officer, Ministry of Health; G. F. McCleary, M.D., and W. T. Fitzgerald, Joint Secretaries of the Ministry of Health.

In the consideration of the Welsh applications the Committee was assisted, by desire of the Minister, by Dr. Meredith Richards.

The total number of applications received was 1,325. Of these, 1,242 were in respect of English and 83 in respect of Welsh appointments (certain candidates applying under both heads), 159 of those applying for English appointments and 14 of those applying for Welsh appointments were selected for interview. In the selection of suitable candidates the Committee carefully weighed in regard to each the evidence of his qualifications in the following respects without attaching preponderating weight to any particular set of considerations:—

(*a*) Medical degrees and diplomas, academic distinctions, and early clinical experience in resident appointments at hospitals. (*b*) General clinical experience (1) in general practice, especially practice among the industrial population in England or Wales, and (2) in other fields of medical practice, whether in civil life or as a medical officer of His Majesty's Forces. (*c*) Experience as a medical referee, whether in connection with National Insurance, Workmen's Compensation, the work of the Ministry of Pensions, or otherwise. (*d*) Experience of National Health Insurance administration, as a member of an Insurance Committee or otherwise, and other administrative experience—*e.g.*, in public appointments or in military service. (*e*) Professional and public reputation in the district in which the candidate practised. (*f*) Personal characteristics, including presence, force of character, and tact.

Though generally it was decided that candidates should be between forty and fifty-five years of age, a few younger or older candidates were included on account of exceptional qualifications among those recommended. In the result the Committee submitted to the Minister the names of fifty-five candidates suitable for appointment in England and of eight candidates suitable for appointment in Wales.

* Nominated by the Civil Service Commissioners.

### The Minister's Selection.

The Minister has decided, with the concurrence of the Treasury, that thirty whole-time officers should be appointed for England and three for Wales at the inception of the scheme. After careful consideration of the recommendations of the Committee he has appointed (subject in the case of officers eligible for establishment to the usual Civil Service Medical Examination) **the following Medical Officers for England:—**

G. Ashton, 2 Palatine Road, Withington, Manchester; S. A. Bontor, Great Berkhamstead; T. L. Bunting, Scotswood-on-Tyne; T. M. Carter, Burnley, Westbury-on-Trym, Bristol; W. Davidson, 91 Wimborne Road, Bournemouth; W. Duncan, 169 Ashley Gardens, S.W. 1; J. Gray Duncanson, 1 Ranelagh Villas, Hove, Sussex; G. W. Eustace, Maltravers Street, Arundel; A. Heath, 66 Otley Road, Harrogate; A. Linnell, Paulerspury, Towcester; M. G. McElligott, 23 Standish Gate, Wigan; W. Moir, Belgrave Square, Darwen; D. G. Newton, 7 Gladstone Road, Sheffield; J. Orton, Great Heath House, Coventry; R. Paterson, 247 Boundary Street, Liverpool; B. A. Richmond, 34 Bromley Road, Catford, S.E.; W. Rigby, 16 St. Alban's Place, Blackburn; B. M. H. Rogers, 14 Mortimer Road, Clifton, Bristol; J. Dill Russell, Osman House, Fortis Green, E. Finchley, N.; H. L. Rutter, 31 West Parade, Newcastle-on-Tyne; E. W. Selby, 13 Hall Gate, Doncaster; H. Sinigar, Western Road, Southall, Middlesex; G. K. Smiley, 20 Kedleston Road, Derby; M. R. Taylor, 64 Churchfield Road, Acton; J. F. Walker, Rocklands, Clifton Terrace, Southend-on-Sea; H. A. Whitcombe, Locharwoods, Formby, Liverpool; E. H. Willock, 91 London Road, Croydon; C. R. Wood, 75 Langdale Road, Hove; A. Young, 43 Collegiate Crescent, Sheffield. There is one vacancy still under consideration.

**For Wales the following have been appointed:—**

J. Evans, Bron Menai, Carnarvon; D. N. Morgan, Gilfach Goch, near Bridgend; T. R. Llewellyn, Penygraig, Glam.

### Divisional Medical Officers.

For England it has also been decided to appoint four Medical Officers of higher grade, who will be designated Divisional Medical Officers, and will superintend the work of the whole-time and part-time Medical Officers in the Divisions assigned to them respectively. The following have been appointed to these posts:—

R. E. Crosse, 68 Nightingale Lane, S.W.; A. Fulton, Armoy, Old Basford, Nottingham; C. H. Milburn, 13 York Place, Harrogate; H. J. Neilson, Shandon, St. George's Hill, Weybridge.

In Wales the Senior Medical Officer of the Ministry for that country will discharge the functions, as regards the outdoor medical staff, which are discharged in England by the Divisional Medical Officers.

